# Investigating the biological significance of the TCM principle “promoting urination to regulate bowel movements” through the influence of the intestinal microbiota and their metabolites on the renal-intestinal axis

**DOI:** 10.3389/fcimb.2024.1523708

**Published:** 2025-01-10

**Authors:** Donglin Yu, Junxi Shen, Liwen Li, Qi Long, Shiqin Xie, Mengsi Zhou, Qianghong Tian, Ying Cai

**Affiliations:** College of Chinese Medicine, Hunan University of Chinese Medicine, Changsha, Hunan, China

**Keywords:** renal-intestinal axis, promoting urination to regulate bowel movements, diarrhea, intestinal microbiota, TCM, fluid metabolism

## Abstract

Treatment methods in traditional Chinese medicine (TCM) are foundational to their theoretical, methodological, formulaic, and pharmacological systems, significantly contributing to syndrome differentiation and therapy. The principle of “promoting urination to regulate bowel movements” is a common therapeutic approach in TCM. The core concept is “promoting the dispersion and drainage of water dampness, regulating urination to relieve diarrhea,” yet its scientific underpinning remains unclear. Modern medical treatment for watery diarrhea primarily focuses on electrolyte replenishment, as diuretics may lead to dehydration and other side effects. Some reports suggest that this TCM approach lacks scientific validity. Microecology, an area associated with the origins of TCM, is closely related to the development, diagnosis, and treatment of diarrhea. The renal-intestinal axis offers a molecular biological basis for examining associated pathological mechanisms, advancing therapeutic targets such as “treating the intestine to address kidney issues” and highlighting the interactions within the “renal-intestinal microbiota-liquid metabolism” framework, thus providing an endogenous mechanism to support “treating the intestine through the kidney.” An increasing number of studies have shown that the intestinal microbiota and its metabolites, as unique mediators, are involved in the physiological and pathological changes of the body. Therefore, this study explores the relationship between fluid metabolism and diarrhea from the perspective of the intestinal microbiota and its metabolites, aiming to elucidate the biological mechanisms underlying the “promoting urination to regulate bowel movements” therapeutic approach and to clarify the scientific basis for treating diarrhea via the renal-intestinal axis. This research provides new insights for the study of TCM microbiology.

## 1 Introduction

With growing concerns about environmental issues and food hygiene, the incidence of diarrhea has been increasing. Research estimates that approximately 1.7 billion cases occur globally each year, making diarrhea the third leading cause of death among infants and young children and posing substantial health risks and economic burdens (Mallier et al., 2023; Guo et al., 2024b). As a common global public health issue, diarrhea significantly impacts patients’ quality of life and imposes an economic burden. Consequently, the prevention and management of diarrhea are urgently needed. Although the concept of the “renal-intestinal axis” does not exist in TCM, the principle of “promoting urination to regulate bowel movements” is a TCM treatment approach addressing dysfunction of the small intestine, where liquid metabolism shifts to the large intestine, leading to excessive dampness and diarrhea. According to TCM theory, the kidney governs water and fluids and opens into the urethra and anus, making them closely associated with fluid imbalance and diarrhea. To some extent, the kidney and intestine exhibit synergistic or inhibitory effects in maintaining fluid homeostasis and excreting waste products. In Western medicine, chronic enteritis, malabsorption syndrome, diarrhea-predominant irritable bowel syndrome, functional diarrhea, and diseases associated with endocrine and metabolic disorders caused by digestive organ dysfunction or organic lesions can all be addressed with TCM-based syndrome differentiation and treatment for diarrhea (Zhang et al., 2017). With advancements in research, diarrhea and intestinal microbiota dysbiosis have been shown to exhibit a bidirectional relationship. Bioactive metabolites can directly or indirectly influence the physiological functions of the host (Meng et al., 2020; Zhu JY. et al., 2023). Several studies have confirmed that certain metabolites can activate phosphorylation and antigen expression, contributing to the development of kidney disease. However, it remains unclear whether diarrhea induced by intestinal fluid leakage is related to the TCM concept of “kidney diarrhea”. The renal−intestinal axis may provide an opportunity to explain the relationship between the kidneys and intestinal tissues. This study is based on the TCM principle of “promoting urination to regulate bowel movements” and investigates the influence of the intestinal microbiota and its metabolites on the renal-intestinal axis. It links changes in the intestinal and renal levels, as well as fluid metabolism indicators, to explain the scientific basis of treating diarrhea through fluid metabolism. This research enriches the scientific understanding of the principle of “promoting urination to regulate bowel movements” and promotes the modernization of TCM treatment methods.

## 2 Relationships between intestinal microecology and diarrhea

### 2.1 Intestinal microbiota and diarrhea

As a potential organ, the intestinal microbiota emerges from the interplay among the intestinal environment, lifestyle, and dietary habits, with intestinal microecology closely tied to the diversity and relative stability of microbial communities (He et al., 2020). Diarrhea, a gastrointestinal functional disorder, is often associated with dysbiosis of the intestinal microbiota, which further exacerbates diarrhea, creating a vicious cycle. According to the *Expert Consensus on TCM Diagnosis and Treatment of Diarrhea* (2017), diarrhea can be classified into six patterns: cold dampness affecting the spleen pattern, damp heat in the intestine pattern, syndrome of retention of food in the stomach, spleen qi deficiency pattern, kidney yang deficiency pattern and liver qi affecting the spleen pattern. [see (Guo et al., 2024b)]. Studies indicate that dysbiosis patterns vary across TCM syndrome types of diarrhea, with distinct treatment methods exerting unique regulatory effects on dysbiosis. For example, in the context of diarrhea associated with cold dampness affecting the spleen pattern, the relative abundances of *Lactobacillus*, *Candidatus Arthromitus*, and *Enterobacter* significantly increased (*p*<0.05). In contrast, diarrhea related to damp heat in the intestine was characterized by increased relative abundances of *Neisseria* and *Capnocytophaga* (*p*<0.05), whereas the relative abundance of *Prevotella* significantly decreased (*p*<0.05). Research on *Huo Xiang Zheng Qi San* and *Ge Gen Qin Lian Tang* revealed differing dominant bacterial species in the intestinal contents and intestinal mucosa, underscoring the complex relationship between the intestinal microbiota composition and diarrhea in terms of both microbial species and abundance (Li et al., 2022). A model of liver qi affecting the spleen pattern with diarrhea induced by Sennae Folium and tail clipping leads to increased intestinal microbial diversity and significant enrichment of pathogenic bacteria such as *Bacteroides vulgatus*, *Helicobacter ganmani*, *Staphylococcus lentus*, and *Lactobacillus murinus* (Yuan et al., 2020). In a kidney yang deficiency pattern diarrhea model induced by adenine combined with Sennae Folium gavage, microbial populations in the cecum, including *Alistipes*, *Enterorhabdus*, and *Desulfovibrio*, are significantly reduced, whereas *Bacteroides* and *[Ruminococcus]_torques_group* are substantially increased (Zhou et al., 2023; Guo et al., 2024a; Zhou et al., 2024). Additionally, distinctive microbial taxa characterize the diarrhea groups for the syndrome of retention of food in the stomach and spleen qi deficiency patterns, represented by the enrichment of *Akkermansiaceae*, *Erysipelotrichia*, and *Ruminococcaceae* and by *Staphylococcus* and *Thermoactinomyces*, respectively (You et al., 2021; Liu J. et al., 2023).

### 2.2 Microbial metabolites and diarrhea

**Figure 1:**
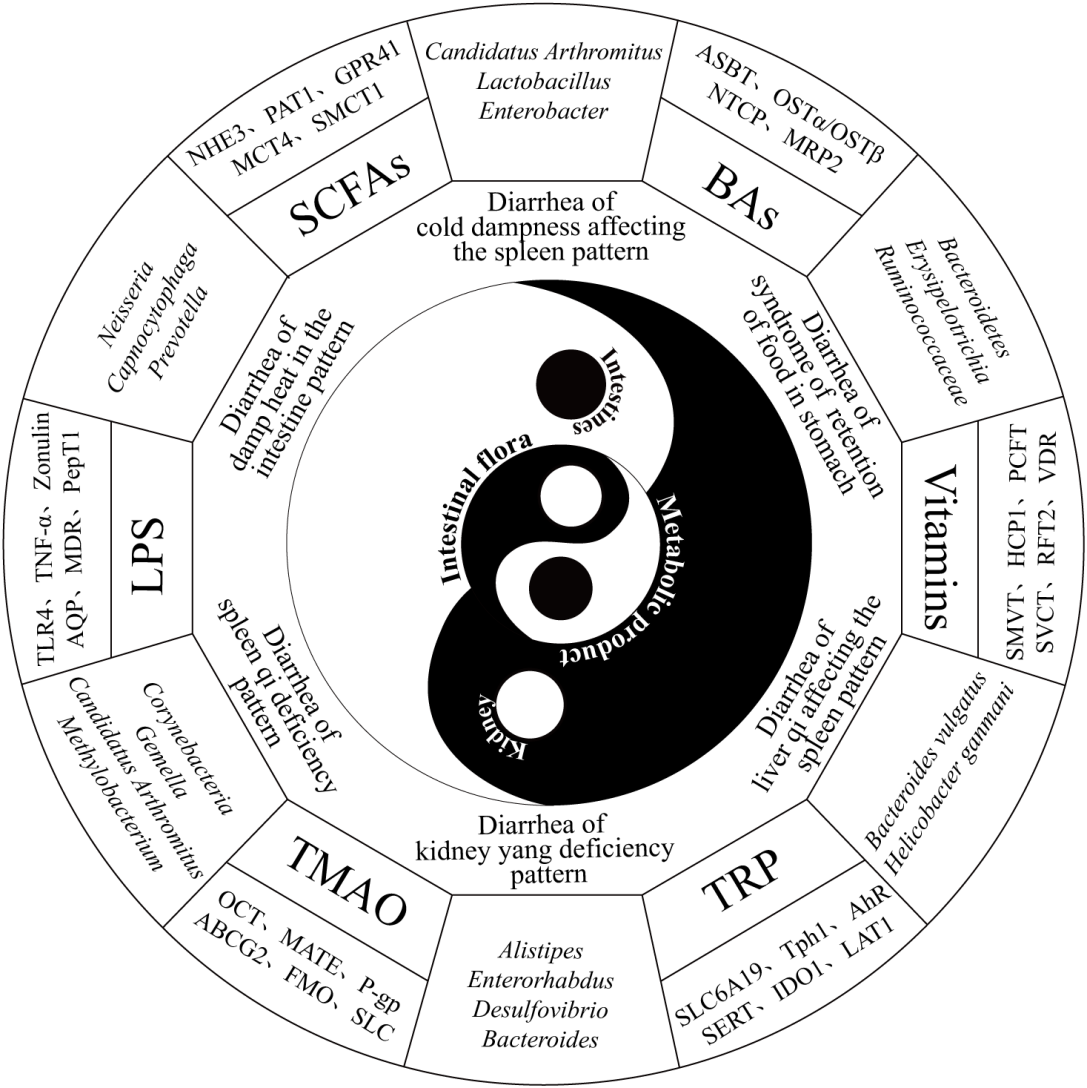
Diagram illustrating the relationships among the renal−intestinal axis, the intestinal microbiota, and the six major types of diarrhea syndromes, along with representative channel proteins and transporters. The diagram is organized from the inside out, representing the intestinal microbiota, renal-intestinal axis, six major TCM diarrhea syndromes, characteristic microbial changes, key microbial metabolites, and corresponding transporter proteins.

Microbial metabolites are small molecules produced through host−microbiota interactions that play critical roles in modulating resistance to pathogenic intestinal bacteria and mediating host−microbe interactions (Nicolas and Chang, 2019). The primary microbial metabolites include short-chain fatty acids (SCFAs), tryptophan (TRP), lipopolysaccharides (LPS), bile acids (BAs), and trimethylamine N-oxide (TMAO); abnormal levels of these metabolites are closely associated with diarrhea (**Figure 1**). SCFAs such as acetate, propionate, and butyrate are recognized by the GPR41/43 and GPR109A receptors, which mediate the activation of NOD-like receptor protein 3 (NLRP3) and hypoxia-inducible factor (HIF). This activation enhances intestinal barrier function, thereby improving gastrointestinal disorders (Macia et al., 2015; Di et al., 2024). Substrate transporters, such as MCT1 and SMCT1, facilitate the absorption of SCFAs and enhance cellular metabolism. Furthermore, SCFAs can activate immune-regulated cascade responses through GPCRs. Transgenic mouse models have confirmed the critical role of GPCRs in intestinal inflammation (Parada, Venegas. et al., 2019). TRP metabolism produces indole-containing metabolites that activate aryl hydrocarbon receptors (AHRs) to regulate immune responses. Studies have shown that *Card9*-deficient mice, which lack protective alleles against inflammatory bowel disease (IBD), display increased susceptibility to colitis and impaired TRP metabolism by the intestinal microbiota (Lamas et al., 2016). As a nonspecific immunogen, LPS stimulates the immune system, disrupts the intestinal mucosal barrier, and enters the bloodstream, where it promotes inflammatory responses (Candelli et al., 2021). The NF-κB signaling mechanism mediates the production of proinflammatory cytokines and chemokines in response to the LPS-LBP-CD14-MD2 complex. This process is involved in maintaining cellular homeostasis and plays a critical role in its regulation (Citronberg et al., 2018; Wang X. et al., 2020). TMAO modulates inflammatory responses, regulates vascular endothelial growth factor, inhibits nitric oxide synthesis, and interferes with cellular signaling, all of which are linked to microbiota dysbiosis and intestinal mucosal barrier dysfunction in IBD (Papa et al., 2023). Organic cation transporter 2 (OCT2) is a key protein that facilitates the uptake of TMAO into renal tubular cells. Experiments have shown that knockout of OCT1/2 in mice leads to elevated plasma TMAO levels and reduced renal retention (Teft et al., 2017). Excess BAs in the colon induce alterations in chloride ion secretion, water−electrolyte balance, and intestinal motility. In patients with diarrhea-predominant irritable bowel syndrome (IBS-D), BA metabolism abnormalities are associated with diarrhea and visceral hypersensitivity, as evidenced by reduced *Ruminococcaceae* abundance (Hou et al., 2022).

Diarrhea is accompanied by an increase in stool water content, resulting in loose or watery stools. Diarrhea caused by impaired intestinal epithelial fluid transport and absorption usually involves both paracellular and transcellular pathways. The paracellular pathway is mediated by the spaces between tight junctions, whereas the transcellular pathway relies on passive diffusion, cotransport, and active transport (Zhu C. et al., 2023). Studies have shown that HgCl2 or CuSO4 can inhibit the water transport and metabolic function of colon AQP3, leading to an increase in the stool water content (Ikarashi et al., 2012). Additionally, in patients with bile acid malabsorption, reduced mRNA and protein levels of AQPs have been observed (Ikarashi et al., 2019), suggesting that proteins or ion channels involved in cotransport or active transport potentially play a role in fluid homeostasis and intestinal health. Therefore, further research is needed to explore the potential mechanisms by which ion channels or transporters regulate diarrhea through intestinal microbiota modulation.

## 3 Causal relationship between liquid metabolism and diarrhea

### 3.1 Regulating urination to relieve diarrhea in TCM

In TCM, dampness is categorized as either internal or external. Internal dampness generally results from deficiency of the spleen and kidney yang or impaired Sanjiao function, leading to water retention, whereas external dampness refers to the effects of humid environments and climates on the skin. Owing to the close relationship of the spleen with dampness, external dampness can weaken the spleen yang. The spleen is responsible for transforming food and fluid, but when dampness hinders its functions, internal dampness accumulates. Water dampness retained under the skin manifests as edema, whereas in the intestine, it leads to diarrhea (Zhang et al., 2023). “Promoting urination to regulate bowel movements” is a common TCM approach for treating watery diarrhea by reducing dampness. This principle primarily arises from ancient insights into the physiological interactions between the small intestine, bladder, kidney, and large intestine (Zhang and Li, 2017; Ling et al., 2023). First, liquid flows from the lower end of the small intestine into the bladder, where fluid transformation occurs to form urine. Pathologically, the small intestine and bladder influence each other. This is evidenced by the statement “bladder heat moves to the small intestine…” from *Sheng Ji Zong Lu* (Song Dynasty) and the discussion of “small intestine diarrhea” in *Nan Jing Zheng Yi* (Qing Dynasty). Second, the large intestine absorbs residual liquid from the small intestine, drying waste before excretion. Disruption of the small intestine’s function in separating clear from turbid fluids or disorders in the metabolism of bodily fluids can lead to dysfunction in the propulsion function of the large intestine, thereby affecting normal urination and defecation. Finally, the kidney and gastrointestinal organs represent the congenital and acquired foundations, respectively: urination and defecation depend on the kidney, whereas food digestion and absorption require coordinated functions between the kidney and stomach (as shown in **Table 1**). Zhang Jiebin, a prominent physician of the Ming Dynasty, attributed the etiology and pathogenesis of diarrhea to pathogenic factors, including cold evils, fire-heat evils, and dampness evils, emphasizing their close association with the large intestine, small intestine, and Mingmen (Kidney) (Xie et al., 2024). On the one hand, “Promoting urination to regulate bowel movements” emphasizes promoting diuresis to restore the physiological functions of the small intestine and kidney, encouraging fluid absorption in the bladder. Increased urination reduces the liquid content in the stool, resulting in a more solid consistency. On the other hand, it means expelling dampness and pathogenic factors through diuresis and is not limited to promoting urination. On this basis, therapeutic methods such as tonifying the spleen, clearing summer heat, warming the kidney, warming the liver, and using sweating and antidiarrheal therapies are all extensions of this treatment approach (Wu LX. et al., 2022).

**Table 1:** Relationships between the renal-intestinal axis, intestinal microbiota, and metabolites and the TCM approach of “promoting urination to regulate bowel movements.”.

TCM	Disease	Research subject	Intestinal microbiota and metabolites	Impact on intestine/kidney level	Liquid and electrolyte indicators	Literature
Modified Ginseng and Wu Mei Decoction	Diarrhea	SD Rats	Regulates *Bacteroides*, *Firmicutes*, *Lactobacillus*, and *Bifidobacterium*; SCFAs are the main products of interactive covariati-on	Decreased D-Lactate and DAO levels after administration; upregulation of Occludin and ZO-1 expression levels	Increased Na^+^, K^+^, Ca^2+^ levels in the Chinese medicine group	(Guan, 2021; Guan et al., 2021)
Huo Xiang Zheng Qi San	Diarrhea due to Cold Dampness Obstructing the Spleen	KM Mice	Restores the quantity of Lactobacillus reuteri and *Lactobacillus casei*; decreases Clostridium ND2 abundance; reduces intestinal lactase activity and increases intestinal mucosal sucrase and lactase activity	Improves intestinal immune response in mice, inhibits inflammatory expression, repairs intestinal barrier integrity	Improvem-ent in loose or watery stools	(Wu et al., 2024)
Qi Wei Bai Zhu San	Antibiotic-associated Diarrhea	KM Mice	Restores abundance of *Escherichia_Shigella*, *Bacteroides*, *Faecalibacterium*; upregulates acetate, propionate, and butyrate levels; normalizes total SCFAs content	IL-17 promotes T-cell activation and stimulates epithelial cells to produce various cytokines, causing inflammation	Upregulate-s Muc2 content and restores it to normal levels	(Li CR. et al., 2023)
Si Shen Wan	Kidney Yang Deficiency Diarrhea	KM Mice	Significant intergroup differences in *Lactobacillus johnsonii*, *Lactobacillus reuteri*, *Lactobacillus intestinalis*, and *Lactobacillus murinus*	Model group shows glomerular hyperplasia, interstitial edema, congestion, inflammatory cell aggregation, varying degrees of lumen dilation, and edema	Affects Na^+^-K^+^-ATPase and Ca^++2+^-Mg^2+^ enzyme activity	[See (Zhu JY. et al., 2023)]
Xin Jia Wei Ling Tang Combined with Gut Microecological Regulators	Persistent Diarrhea	Human Infants	Differences in cfu/g numbers of *Enterobacteriaceae*, *Enterococci*, *Lactobacilli*, and *Bifidobacteria*	Gut mucosal function indicators (ET, DAO), gastrointestinal function indicators (D-lactate, urine L/M ratio) are lower than before treatment	Inhibition of liquid and electrolyte reabsorptio-n; increased urine volume and Na^+^ excretion	(Liu and Guo, 2020)
Bu Zhong Yi Qi Decoction	Spleen Deficiency Diarrhea	SD Rats	Decreased abundance of beneficial bacteria such as Bifidobacterium and Lactobacillus; increased pathogenic bacteria such as *Enterococcus* and *Bacteroides*	Small intestinal villi are partially curved and deformed; loss of epithelial surface; increased interstitial inflammatory cells; altered urinary D-xylose excretion rate	Increased Na^+^-K^+^-ATPase activity after treating; upregulation of SGLT1,GLUT2,NHE3 protein expression	(Liu et al., 2017; Liu, 2018)
Fu Zi Li Zhong Decoction Combined with Si Shen Wan	Diarrhea-type Irritable Bowel Syndrome	Humans	Increased abundance of beneficial bacteria such as *Clostridia*, *Romboutsia*, and *Turicibacter*; decreased abundance of harmful bacteria such as Mycoplasma and *Proteobacteria*	Increased levels of Ghrelin, SP, and MTL; promotes the expression of Corticotropin-Releasing Factor (CRF) and its receptors	One of the drug components, TPLP, can reduce the excretion of fluids and electrolytes	(Sun, 2023)
Guang Xiang Zhi Li San	Epidemic Diarrhea	Piglets	Changes in average relative abundance of Bacteroidetes; endotoxins affect the phagocytic capacity of lymphocytes and the intestinal immune system	Inhibits intestinal inflammation; varying degrees of changes in intestinal villus length and intestinal wall thickness	Soft, unformed stools, watery diarrhea	(Huang et al., 2017)
Jian Pi Jie Du Decoction	Spleen Deficiency with Dampness in Common Psoriasis	Humans	Significant expression of Bacteroidota and Bifidobacteriales before and after treatment; butyrate, SCFAs, and oligosaccharides affect immune cells and cytokines	Inhibits intestinal flora causing intestinal hemorrhage; inhibits inflammation affecting intestinal barrier	Intestinal goblet cell and villus length associated with certain bacterial populations	(Du et al., 2020; Xu, 2021)
Shen Ling Bai Zhu San	Lard-Induced Fatigue-related Diarrhea	KM Mice	Chinese medicine reverses dysbiosis; *Lactobacillus reuteri*, *Lactobacillus intestinalis*, and *Lactobacillus johnsonii* become dominant, while SCFAs such as acetate, butyrate, and valerate significantly increase	Decreased monolayer columnar epithelial cells and columnar cells; increased cell deformation and shedding	Increased fecal liquid content in the model group; reduced goblet cell numbers	(Qiao et al., 2023; Qiao et al., 2024)

### 3.2 Rehydration therapy for diarrhea in modern medicine

**Figure 2:**
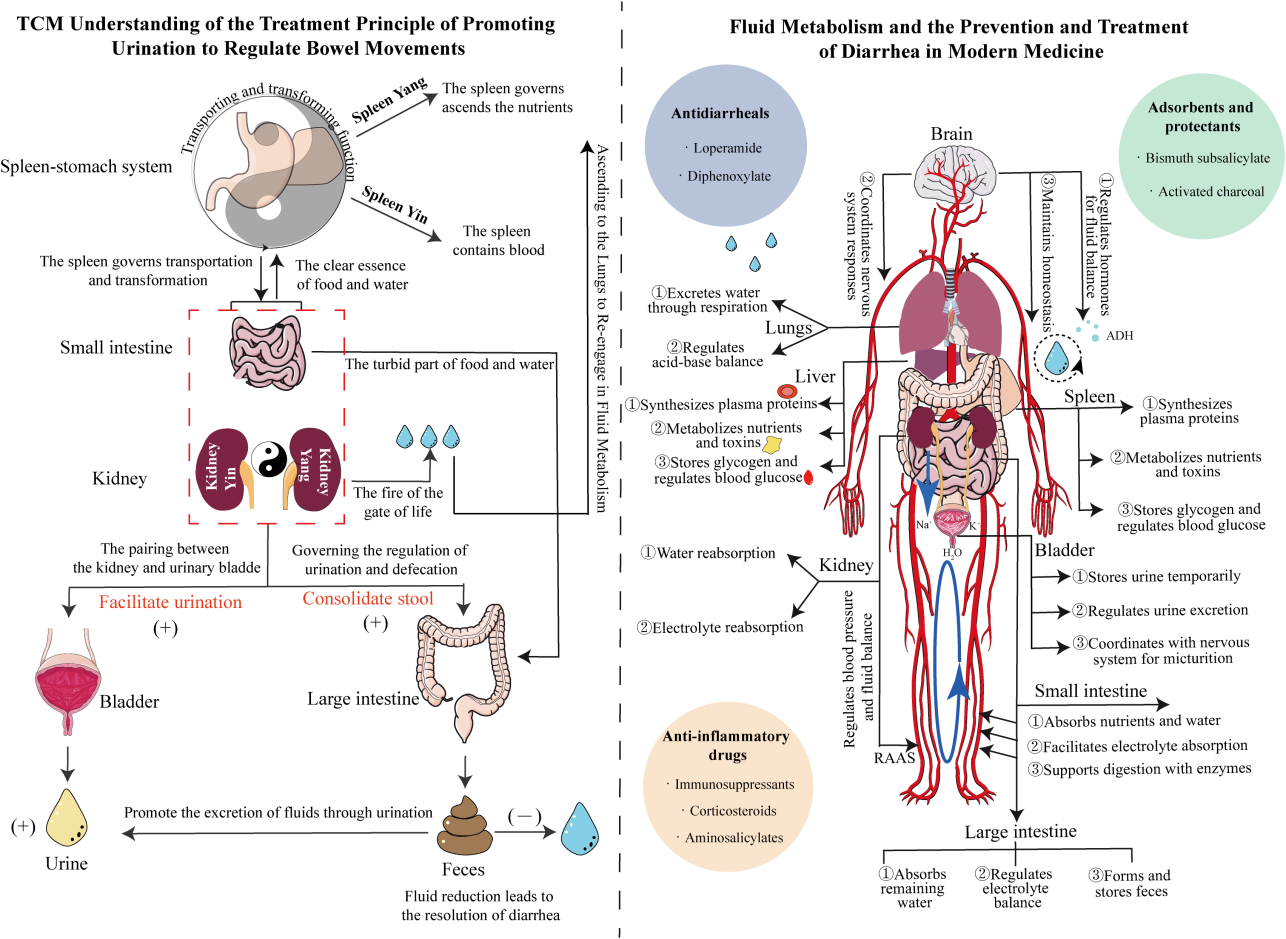
Mechanistic diagram illustrating the differences between TCM’s ability to “regulate urination to relieve diarrhea” and Western medicine’s understanding of fluid metabolism and diarrhea treatment. Left panel: Guided by the theory of Yin-Yang in TCM, the diagram shows the anatomical relationships between the spleen, stomach, small intestine, kidney, bladder, and large intestine, clarifying the significance of promoting water to stop diarrhea. Right panel: Based on internal circulation, the diagram elaborates on the regulation of fluid metabolism by the body’s major organs, emphasizing the importance of replenishing water to treat diarrhea. The blue, green, and orange circles represent antidiarrheal drugs, adsorbents, and anti-inflammatory drugs, respectively.

Modern medicine attributes diarrhea primarily to gastrointestinal motility disorders, intestinal microbiota dysbiosis, and compromised intestinal mucosal barriers, with underlying water−electrolyte imbalances and intestinal smooth muscle dysfunction mediated through neuroendocrine–immune networks (Bai et al., 2021). Intestinal hormones and gut–brain peptides contain endocrine signals. Studies have shown that serotonin (5-HT), corticotropin-releasing factor (CRF), substance P (SP), neuropeptide Y (NPY), and calcitonin gene-related peptide (cGRP) are widely distributed in the central and nervous systems, as well as in various tissues and organs (Wu HM. et al., 2022). After the intraperitoneal injection of CRF in rats, intestinal mucosal permeability increased, and the expression of tight junction (TJ) proteins such as Claudin-1, Occludin, and ZO-1 decreased. Moreover, colonic motility and fluid loss are increased, leading to symptoms of IBS-D, including diarrhea and abdominal pain (Guilarte et al., 2020; Zhuang et al., 2020; Wang et al., 2024). Under normal conditions, the small and large intestines possess a substantial reserve capacity for fluid absorption; diarrhea occurs when stimuli for secretion surpass this anatomical reserve, resulting in secretory diarrhea. The chloride-driven secretion mechanism remains crucial in managing diarrhea and preventing dehydration (Poroca et al., 2020). Studies have shown that diarrhea is associated with the expression of inflammatory factors or neurotransmitters, which stimulate crypt cells to secrete large amounts of water and electrolytes, resulting in increased intestinal luminal volume and accelerated intestinal motility. Cyclic adenosine monophosphate (cAMP) is involved in the secretion of water and electrolytes by crypt cells and affects the absorption of Na^+^ and Cl^-^ in the villous epithelium, leading to significant disturbances in water and electrolyte metabolism, manifesting as watery diarrhea (Keely and Barrett, 2022; Zhao et al., 2022). Fluid therapy is currently the best and fastest treatment for managing hypovolemia or gastrointestinal diseases, such as dehydration caused by severe diarrhea (Tello and Perez-Freytes, 2017). Representative oral rehydration solutions and hypotonic fluids primarily contain sodium, potassium, and glucose to increase blood volume and improve circulation and renal function (Chen et al., 2018). Therefore, modern medical approaches for treating diarrhea prioritize assessing hydration status and implementing rehydration therapy to counteract the adverse effects of dehydration (**Figure 2**).

## 4 Role of the renal-intestinal-microbiota axis in fluid metabolism

### 4.1 The renal-intestinal axis

The renal-intestinal axis theory, initially proposed by Meijers, introduced the concept of the “chronic kidney disease-colon axis,” which emphasizes the pathogenic interactions between the intestinal microbiota and kidney diseases (Li JQ. et al., 2023). This axis comprises a complex network involving the central nervous system, autonomic nervous system, enteric nervous system, hypothalamic−pituitary−adrenal (HPA) axis, and sympathetic−adrenal axis. Research suggests that the kidney and intestine communicate through neurotransmitters, neurotrophic factors, the renin−angiotensin system, and the sympathetic−adrenal axis, establishing reciprocal feedback loops (Han et al., 2023). Renin secreted by juxtaglomerular cells is metabolized by angiotensin-converting enzyme, ultimately transforming into angiotensin II (Ang II). Ang II constricts the afferent and efferent arterioles of the kidney, inhibits mesangial cells, reduces medullary blood flow, and promotes water and sodium reabsorption in the renal tubules (Zhang and Luan, 2024). While TCM does not have the concept of the renal-intestinal axis, it posits that the kidney, large intestine, small intestine, and bladder all belong to the lower burner (lower abdomen). The functions of digestion, absorption, immune modulation, and spleen transportation and transformation are closely related to the formation of urine and stool. The kidney, being the “water organ”, governs and regulates fluid metabolism. One of the causes of diarrhea in kidney yang deficiency syndrome is insufficient kidney yang, leading to failure to warm the earth (spleen), causing water retention in the small intestine (Li XY. et al., 2023). Additionally, the kidney opens into the urethra and the anus and governs water and fluids. Dysfunction in both kidney and intestinal functions, leading to disordered fluid metabolism in the lower burner, may also be a major cause of abnormal urination and defecation. TCM views the kidney as central to liquid metabolism, with kidney-yang deficiency-induced diarrhea resulting from insufficient yang warmth to support digestion, thereby leading to fluid accumulation in the intestine. Consequently, the renal-intestinal axis theory offers a scientific basis for managing diarrhea from the perspective of fluid metabolism.

### 4.2 Dynamic feedback of the renal-intestinal axis on fluid metabolism associated with intestinal microbiota

Intestinal microbiota-mediated signaling pathways play crucial roles in the regulation of diarrhea within the renal-intestinal axis. The relationship between the intestinal microbiota and bodily systems is influenced by external factors such as diet and medication. Chronic kidney disease (CKD) alters the composition and metabolic activity of the intestinal microbiota, damaging intestinal barrier function and allowing endogenous bacterial metabolites, cell wall components, and viable bacteria to enter systemic circulation (Lange et al., 2016; Rysz et al., 2021). This pathological process inhibits the expression of mucin Muc2 and the tight junction proteins ZO-1 and Claudin-1, indirectly affecting the regulation of water, Na^+^, and K^+^ by mucosal epithelial transporters and ion channels. Research indicates that microbial fermentation of proteins in the intestine produces uremic solutes, which may exacerbate kidney inflammation. Increasing the intake of probiotics or synbiotics can alleviate the detrimental effects of dysbiosis, enhance the immune response, protect the intestinal barrier, and reduce uremic symptoms (Rossi et al., 2016). Therefore, the intestinal microbiota may serve as a new target for the treatment of kidney diseases. Moreover, certain intestinal microbes can act as “signal sources” for both the kidney and intestine, participating in both physiological and pathological regulation. CKD patients exhibit microbial trends similar to those observed in IBD patients, with elevated levels of *Bacteroides*, *Escherichia*, *Enterococcus*, and *Clostridium* species, alongside decreased *Lactobacillus*, suggesting the involvement of the intestinal microbiota in renal-intestinal feedback, where each microbe exhibits distinct patterns within the axis system (Cooper et al., 2021). Additionally, the renal-intestinal-microbiota axis is involved in the generation, metabolism, and excretion of water and fluids. Uremic toxin indoxyl sulfate (IS), derived from gut *Escherichia coli*, regulates the IRF1-DRP1 pathway, disrupting the intestinal mucosal barrier, blocking mitochondrial autophagy, and triggering mitochondrial damage. Mitochondrial damage is common in impaired organelles in the kidney, intestine, and other organs in chronic kidney disease and represents a potential therapeutic target (Huang et al., 2020a; Huang et al., 2020b; Huang et al., 2022). Furthermore, the kidney requires high levels of mitochondrial activity to support water filtration, reabsorption, and secretion functions. Mitochondrial dysfunction leads to reduced ATP production or accumulation of reactive oxygen species (ROS), which diminishes the ability of renal tubules to reabsorb water and electrolytes, resulting in disturbances in fluid metabolism (Ho and Shirakawa, 2022; San-Millán, 2023).

### 4.3 Key role of intestinal microbial metabolites in the renal-intestinal axis and fluid metabolism

The intestinal microbiota produces many metabolites that promote host physiological functions, acting as signaling molecules and substrates for metabolic reactions within the host. Currently, these metabolites have become potential microbial targets for new therapies (Descamps et al., 2019; Krautkramer et al., 2021). Studies have shown that the effects of individual microbial compounds are uncertain and depend on factors such as the type of tissue affected, metabolic state, dietary environment, and baseline circulating levels (Deschasaux et al., 2018; Jha et al., 2018; Vangay et al., 2018). Moreover, the production of microbial metabolites is determined by dietary substrates and individual differences between populations. Preliminary studies have also demonstrated that the intragastric administration of lard combined with a multiplatform water environment can induce changes in bile acid levels in mice (Yu et al., 2024). Moreover, the interaction between microbial metabolites and the host microenvironment also shows cyclical variations. Substrate utilization affects microbial fermentation, which in turn impacts the production of metabolites, such as SCFAs, that intervene in the body’s pH, ultimately leading to changes in the microbial cLPS community structure and function (Desai et al., 2016; Makki et al., 2018). This study selected representative microbial metabolites, such as SCFAs, TMAO, BAs, LPS, and AAs, to explore their associations with fluid metabolism-related transporter proteins, hormone levels, and the dynamic feedback of the renal-intestinal axis in influencing diarrhea.

#### 4.3.1 SCFAs-mediated regulation of intestinal barrier and renal inflammation

SCFAs are a class of low-carbon saturated fatty acids that are derived primarily from the fermentation of undigested carbohydrates by the intestinal microbiota and are associated with intestinal inflammation, colorectal cancer, and metabolic diseases (Wang and Bao, 2020). On the one hand, SCFAs promote intestinal barrier function, enhance tight junction and mucus secretion in intestinal epithelial cells, activate GPR41 and GPR43 receptors, and inhibit histone deacetylases (HDACs). Studies have shown that monocarboxylate transporter 1 (MCT1/SLC16A1) and sodium-coupled monocarboxylate transporter 1 (SMCT1/SLC5A8) enhance the absorption of acetate, propionate, and butyrate, promoting cellular metabolism [see (Parada, Venegas. et al., 2019)]. Butyrate induces the expression of genes encoding tight junction (TJ) components or activates other transcription factors (such as STAT3 and SP1), reorganizing proteins to facilitate the epithelial barrier’s regulation of water and fluid (Kelly et al., 2015). On the other hand, SCFAs can suppress renal inflammation, inhibit oxidative stress, regulate autophagy, and improve energy metabolism. Clinical trials have shown that in CKD stage 5 patients, the abundance of SCFA-producing bacteria, such as *Prevotella* and *Roseburia*, decreases, and the abundance of these bacteria is negatively correlated with kidney function (Wang XF. et al., 2020). The administration of sodium acetate and sodium butyrate or GPR43 agonists significantly inhibits hyperglycemia and LPS-induced mesangial cell proliferation, reverses the production of ROS and malondialdehyde, and increases superoxide dismutase levels, exerting antioxidant effects in diabetic nephropathy. SCFAs binding to GPR43 suppresses the degradation of IκBα and the phosphorylation of NF-κB p65 in mesangial cells under hyperglycemic conditions, reducing ROS and malondialdehyde production and effectively improving renal tissue pathology (Huang W. et al., 2020; Zhong and Cai, 2021). Interestingly, SCFAs can also enter immune cells through transporters such as MCT1/4 and SMCT11/2, exerting anti-inflammatory effects, alleviating the damage caused by inflammatory mediators to barrier function, and potentially reducing fluid permeability (Yao et al., 2022).

#### 4.3.2 Intestine-derived TMAO and its dual role in renal health and fluid dynamics

TMAO is a metabolite formed from the oxidation of trimethylamine (TMA) by intestinal bacteria in the liver. The levels of such metabolites are often used as biomarkers for the prognosis of patients with colitis (Jalandra et al., 2021). Studies indicate that TMAO induces inflammation and triggers colon cancer through various mechanisms, including DNA damage, oxidative stress, and disruption of protein folding (Chan et al., 2019). As a potential biomarker, TMAO may aid in identifying the progression of CKD. Clinical investigations have shown that plasma TMAO levels in CKD patients are correlated with patient prognosis and survival rates. In animal models, prolonged dietary exposure to high TMAO concentrations leads to renal fibrosis and dysfunction in mice, suggesting that TMAO acts as a mediating factor in the communication between the kidney and intestinal tissues (Tang et al., 2015; Missailidis et al., 2016; Cui et al., 2023). The levels of urease, uricase, para-cresol, and indole-producing bacteria increase in CKD patients and other kidney disease patients, leading to the accumulation of harmful substances in the serum. These patients often take antibiotics and experience dietary changes that exacerbate dysbiosis of the intestinal microbiome (Zheng et al., 2022). Research has shown a positive correlation between TMAO levels and renal function impairment; the potential pathways through which TMAO affects CKD primarily include oxidative stress, inflammation, and endoplasmic reticulum stress (Pan et al., 2023). These metabolic pathways provide a basis for the relationship between TMA/TMAO produced by the intestinal microbiota and renal dysfunction and fibrosis while also establishing a link between TMAO levels and glomerular and kidney injury. Some studies have identified organic cation transporter 2 (OCT2) as an ion transporter located on the basolateral side of renal tubular epithelial cells that mediates the entry of TMAO into tubular cells, whereas various ATP-binding cassette (ABC) transporter proteins facilitate TMAO excretion (Humphreys, 2018; Ye et al., 2022). Recent studies suggest that the increase in blood TMAO in diseases such as diarrhea, which is associated with water and electrolyte imbalance, may correspond to the compensatory response of natriuretic peptide B (BNP) to water and osmotic stress, indicating a potential beneficial role of TMAO in organs under pressure or volume overload (Ufnal and Nowiński, 2019).

#### 4.3.3 Bile acids and their impacts on renal tubular function and fluid regulation

BAs are steroid molecules synthesized from hepatic cholesterol metabolism that are capable of activating nuclear receptors (NRs) and G protein-coupled receptors (GPCRs). As signaling factors, BAs primarily interact with two types of BA receptors—farnesoid X receptor (FXR) and G protein-coupled bile acid receptor 1 (TGR5)—and are involved in the regulation of the intestinal mucosal structure and function (Shi et al., 2023). Bile acid diarrhea (BAD) is a form of chronic diarrhea mediated by bile acids and is associated with changes in intestinal epithelial permeability, TGR5 activity, and 3’,5’-cyclic adenosine monophosphate (cAMP) function. Research has shown that doses of 500 mg or 1000 mg of ursodeoxycholic acid significantly increase the frequency of colonic motility, which is correlated with stool frequency and consistency (Camilleri and BouSaba, 2023). BAs also function as signaling molecules, influencing renal metabolism. Under physiological conditions, epithelial cells of the renal proximal tubules take up BAs via the apical sodium-dependent bile acid transporter (ASBT); however, excess accumulation of BAs in the kidney may lead to bile acid nephropathy. The administration of ASBT inhibitors can increase the urinary excretion of BAs and reduce the body’s BA pool (Ghallab et al., 2024). Bile acid nephropathy (BN) is one of the major causes of acute kidney injury (AKI), leading to renal tubular cell damage, cast formation, progressive tubular dilation, and interstitial fibrosis, thereby affecting the urine concentration and the process of water and electrolyte reabsorption (Fickert and Rosenkranz, 2020). *Lactobacillus* and *Lactobacillus reuteri* are key bacterial genera that encode bile salt hydrolase (BSH), which is involved in BA modification and influences free BA levels in the body. Additionally, bile acids can also directly influence the overall metabolism of microbiota, such as *Listeria monocytogenes* and *Ruminococcaceae*, as well as the overall metabolism of amino acids, nucleotides, and carbohydrates (Li et al., 2017; Xia et al., 2024). In a mouse model of spleen deficiency and dampness syndrome diarrhea, secondary bile acid biosynthesis not only affects gastrointestinal motility and intestinal water secretion but also directly or indirectly influences the intestinal microbiota. The antimicrobial activity of secondary bile acids, which are produced through deconjugation, impacts specific microbial communities in the intestine [see (Wei et al., 2020; Yu et al., 2024)]. Research has revealed that bile acid-mediated bile acid malabsorption syndrome is associated with abnormalities in intracellular mediators, intestinal permeability, water channel proteins, and intestinal and neurosecretory mechanisms. Serotonergic and cholinergic neurons increase fluid and mucus secretion, reduce sodium and water reabsorption, and stimulate colonic motility (Donato et al., 2018; Camilleri and Vijayvargiya, 2020).

#### 4.3.4 Intestine-derived LPS and its systemic effects on renal function and electrolyte homeostasis

LPS is a glycolipid found in the outer membrane of gram-negative bacteria that serves as a barrier against bacterial toxins. LPS is released during bacterial cell death or active proliferation. Studies indicate that LPS binds to the membrane protein CD14 with the assistance of lipopolysaccharide-binding protein (LBP); subsequently, the coreceptor myeloid differentiation protein 2 (MD2) aids in this recognition process. In patients with IBD, such as those with Crohn’s disease (CD), LPS promotes the expression of extracellular matrix protein 1 (ECM-1) in intestinal macrophages, thereby interacting with components of the innate immune system (Heinbockel et al., 2018). Knockout mice lacking the MyD88 gene, which encodes an inflammatory differentiation factor, do not show increased levels of serum inflammatory factors, indicating an antagonistic effect on LPS expression (Shan et al., 2019). In gastrointestinal diseases, tissue damage leads to the release of LPS into the bloodstream, where it circulates and continues to affect kidney physiology as an antigen. In the late stages of CKD, the kidney often exhibits fibrosis; supplementation with *Bacteroides fragilis* can upregulate SGLT2, facilitating the reabsorption of 1,5-anhydroglucitol (1,5-AG) and inhibiting oxidative stress and inflammation. However, LPS can suppress the beneficial effects of *B. fragilis* (Zhou et al., 2022). Moreover, the renin-angiotensin-aldosterone system (RAAS) regulates blood pressure and electrolyte concentrations, modulates inflammation, and maintains immune balance. Studies have shown that LPS intervention affects the RAAS, contributing to the imbalance between the ACE/Ang II and ACE2/Ang1-7 axes. Certain flavonoid compounds can inhibit ACE expression, urinary renin activity, and the excretion of urinary prorenin/renin under LPS stimulation. This effect partially depends on the inhibition of the intrarenal ACE/Ang II axis and the activation of the ACE2/Ang1-7 axis, independent of the systemic RAS (Yang and Xu, 2017; Yi et al., 2023). It has been suggested that the modulation of LPS expression to intervene in RAAS system hormone levels may regulate urination to relieve diarrhea.

#### 4.3.5 Amino acid transporters in renal and intestinal epithelial cells: implications for fluid balance

**Figure 3:**
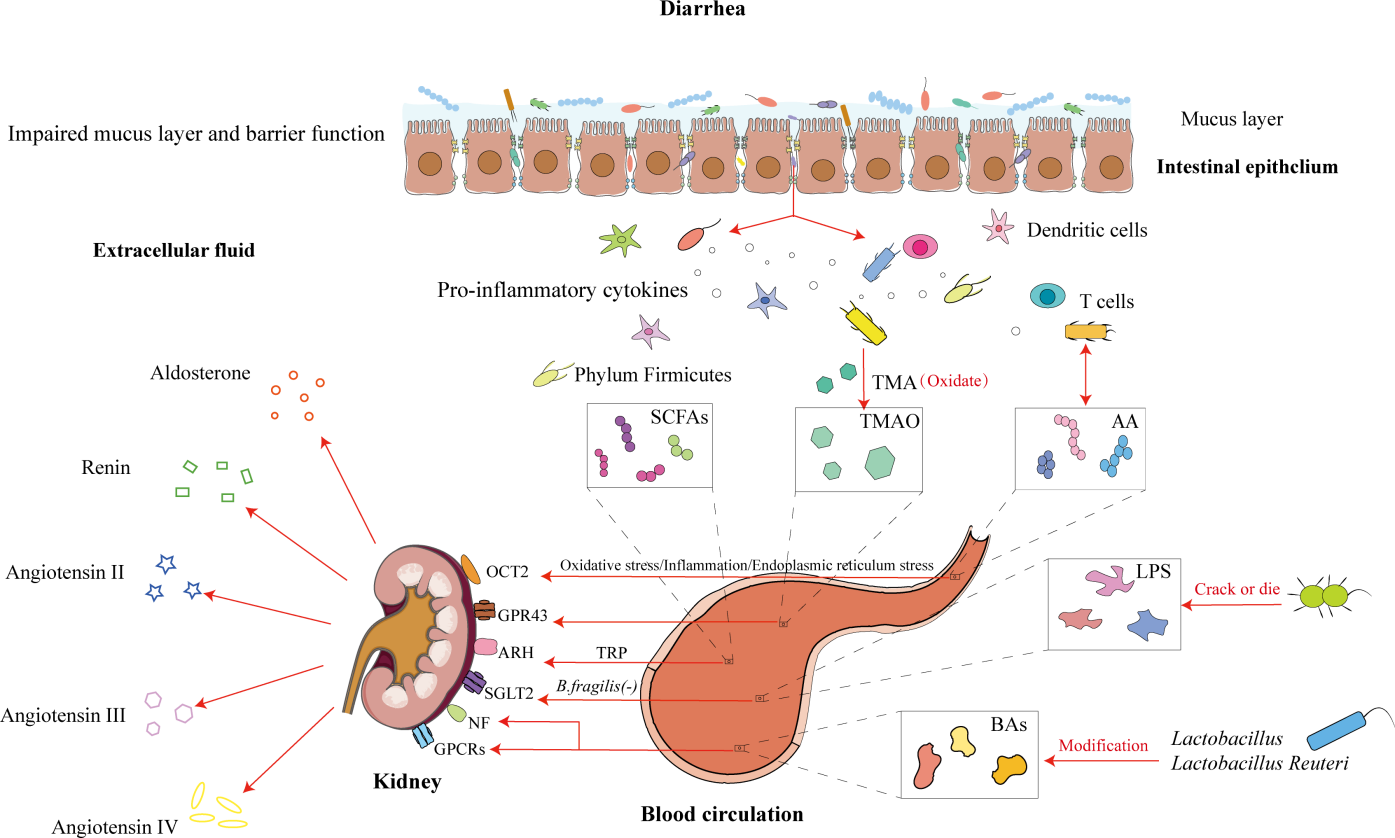
Intestinal microbiota and its metabolites influence the renal-intestinal axis via the modern biological mechanism of promoting urination to regulate bowel movement. Diarrhea causes damage to the intestinal mucosal barrier, with disruption of the mucus layer and an increase in bacterial transmembrane transport. Among these, short-chain fatty acid-producing bacteria, trimethylamine-producing bacteria, amino acid-producing bacteria, lipopolysaccharides, and bile acids, through the blood circulation, act on renal targets such as OCT2, GPR43, and ARH. The kidney regulates hormone levels, which participate in cometabolism and further modulate body fluid metabolism for the treatment of diarrhea.

Amino acids (AAs) serve as intermediates in energy and metabolic pathways, activating signaling networks associated with cell proliferation and growth. The relationship between the intestinal microbiota and amino acid metabolism is mutually regulatory: on the one hand, AAs metabolized by intestinal microbes produce various active metabolites that can either promote or inhibit AA-related microbial populations; on the other hand, the intestinal microbiota supplies essential amino acids for host tissue synthesis (Yang et al., 2013; Guo and Cheng, 2023). Under physiological conditions, intense exercise induces abnormal biochemical demands in the body, such as oxidative stress, intestinal permeability, electrolyte imbalance, and glycogen depletion (Donati Zeppa et al., 2019). *Akkermansia* species can reverse the effects of a high-fat diet by increasing endogenous intestinal cannabinoid levels, suppressing inflammation, enhancing the intestinal barrier, and promoting intestinal peptide secretion to reduce metabolic disorders (Everard et al., 2013). Under pathological conditions, AA can improve abnormal expression of the mTOR pathway in UC, promote protein biosynthesis, and accelerate epithelial cell proliferation. The exercise of the nutrient glutamine enhances the abundance of beneficial bacteria by proliferating epithelial cells and regulating tight junction proteins to suppress colitis and noninfectious diseases (Coqueiro et al., 2019; Tobias et al., 2020). Additionally, AAs affect renal hemodynamics, glomerular blood pressure, glomerular filtration, and structural damage. Interestingly, studies have shown that AAs cotransport with Na^+^ and participate in epithelial transport protein reabsorption. basolateral transporters play important roles in regulating the intracellular concentrations of different AAs. The basolateral AA transporters in the small intestine and proximal renal tubular epithelial cells mainly include Lat2-4F2hc and y+Lat1-4F2hc (Mariotta et al., 2012), whereas T-type amino acid transporter 1 (TAT1) is located between the small intestine and renal tubular absorptive epithelial cells and mediates the transmembrane diffusion of aromatic AAs. The tryptophan (Trp) metabolic pathway, via intestinal microbiota metabolism, directly transforms into indole and its derivatives, with these metabolites acting as ligands for the aryl hydrocarbon receptor (AhR), which activates AhR and promotes the repair of the UC intestinal barrier (Shi et al., 2020). As ligands, tryptophan metabolites can also activate AhR signaling in various diseases, such as inflammation, oxidative stress damage, cancer, age-related diseases, cardiovascular diseases, and chronic kidney disease (Liu et al., 2021; Xue et al., 2023). Currently, AAs are widely involved in digestive, neurological, and urinary system diseases, and elucidating the role of AAs in the renal-intestinal axis may be key to understanding the mechanism by which traditional Chinese medicine promotes urination to regulate bowel movements. (see **Figure 3**).

Current research on the renal-intestinal-microbiota axis has focused primarily on chronic kidney disease, with few studies exploring its role in gastrointestinal disorders characterized by fluid metabolism imbalances. Increased electrolyte secretion and decreased absorption are hallmark features of IBD patients. Notably, cooperative proteins or transport mechanisms, such as NHE, ENaC, and Na^+^/K^+^-ATPase, are associated with elevated serum aldosterone levels in IBD patients (Magalhães et al., 2016). The RAAS plays a role in regulating intestinal transport and barrier functions, as well as in glucose and peptide absorption, gastrointestinal motility, and mesenteric blood flow (Garg et al., 2012; Caruso et al., 2020). Studies indicate that colonic epithelial Occludin and Claudin 8 expression is regulated by aldosterone, suggesting that the RAAS has additional therapeutic potential in IBD (Amasheh et al., 2009). Traditional theories hold that the RAAS system relies on renin and angiotensin to process Ang I, which is subsequently converted by ACE into Ang II, activating aldosterone to regulate blood pressure and circulation. In human IBD and canine chronic inflammatory enteropathy (CIE), the RAAS may involve both classical and alternative pathways, promoting or inhibiting vascular function, inflammation, and fibrosis (Stoyell-Conti et al., 2021; Heilmann et al., 2023). Interestingly, Ang II not only affects intestinal electrolyte absorption by activating ENaC but also has chemotactic effects on inflammatory cells. These findings suggest that the local mucosal microenvironment influences RAAS-mediated expression, whereas inflammatory factors in IBD, such as IL-1, TNF-α, or NF-κB, might activate RAAS components via a positive feedback loop. However, the concentration threshold might also affect the upregulation of electrolyte transport protein transcription (Heilmann et al., 2022; Dengler et al., 2023). Additionally, studies have shown that antibiotic treatment suppresses the intestinal microbiota, such as *Bacteroides fragilis*, leading to increased plasma and urinary aldosterone levels in male and female mice (Moore et al., 2024). These findings highlight the potential of the intestinal microbiota to regulate aldosterone and suggest promising therapeutic avenues for diarrhea through immune and endocrine pathways.

## 5 Key role of the renal-intestinal axis in the treatment principle of “promoting urination to regulate bowel movements”

### 5.1 Theoretical basis of “promoting urination to regulate bowel movements”

**Figure 4:**
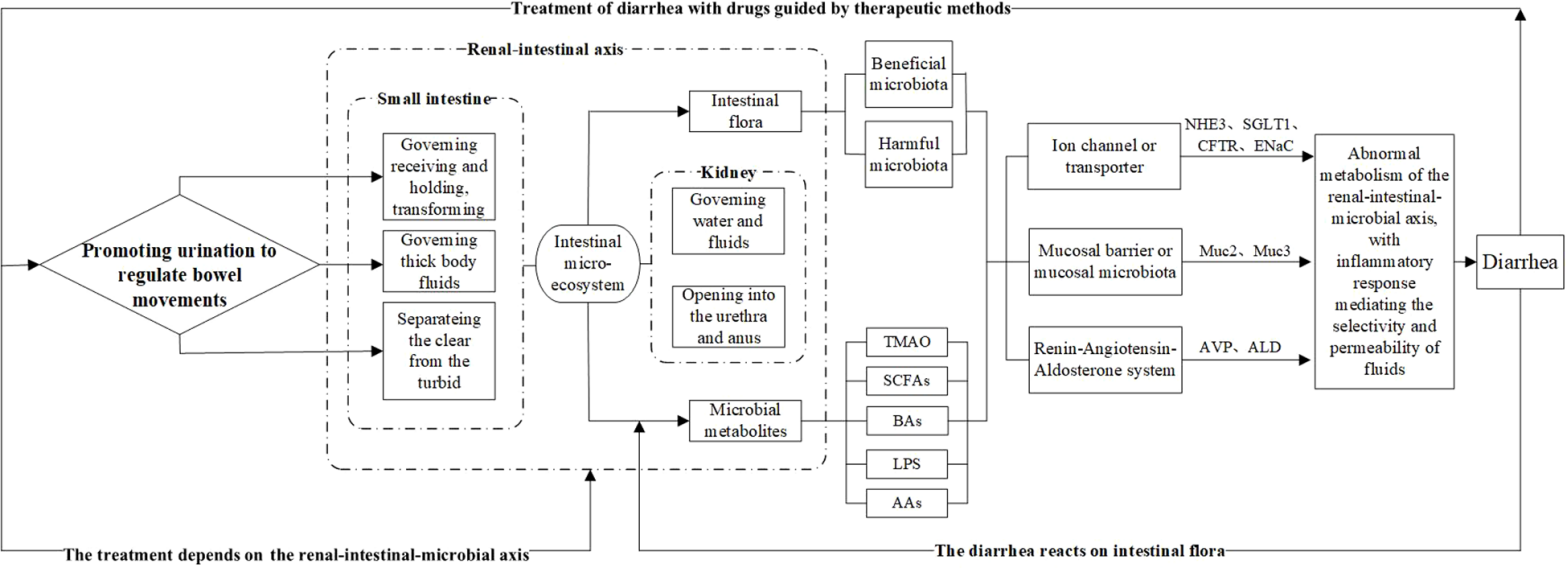
Biological connotation of the TCM treatment principle “Promoting Urination to Regulate Bowel Movements.” The intestinal microbiota and its metabolites serve as intermediary factors, linking the small intestine’s ability to separate clear and turbid fluids, which governs bodily fluids, and the kidney’s ability to regulate excretion and fluid metabolism. These findings provide a modern scientific explanation for the principle of regulating urination to relieve diarrhea. Among them, the intestinal microbiota, represented by beneficial and harmful bacteria, and their metabolic products—such as TMAO, SCFAs, BAs, LPS, and AAs—affect diarrhea development and treatment outcomes. These effects can be evaluated at three levels: water channel proteins and ion channels, the intestinal mucosal barrier, and the RAAS system, with changes in relevant biomarkers after treatment.

The earliest discussion of the physiological and pathological characteristics of the small intestine in TCM was presented by *Huangdi Neijing* (Yellow Emperor’s Inner Canon), which laid the theoretical foundation for the treatment principle of “Promoting Urination to Regulate Bowel Movements”. Later generations of physicians, including Zhang Zhongjing, further developed and summarized this theory, forming a complete theoretical system. TCM suggests that the treatment of diarrhea must address two main issues: first, expelling dampness, and second, strengthening the spleen. Compared with strengthening the spleen, expelling dampness involves giving the pathogenic factors an outlet and guiding the process according to the situation, which is also the fastest method. Clinical studies have also shown that *Wuling San* (a traditional Chinese formula) can inhibit the RAAS, suppress renal tissue water channel proteins, and regulate factors to produce diuretic effects (Ahn et al., 2012; Fang et al., 2025). Therefore, from the perspectives of both TCM and modern medicine, the regulation of fluid metabolism and excretion by the small intestine and kidney is evident (see **Figure 4**).

### 5.2 Relationship between fluid metabolism and intestinal level

One of the functions of the small intestine is to transport electrolytes and fluids between the intestinal lumen and contents, involving both absorption and secretion processes. Under normal conditions, fluid secretion into the intestinal lumen serves to hydrate the mucosa and maintain intestinal barrier function, a process influenced by hormones, neurotransmitters, immune cell mediators, dietary components, and microbes, along with their metabolites [see (Poroca et al., 2020)]. Diarrhea disrupts the homeostatic mechanisms of the small intestine, causing Cl^−^-driven fluid secretion to exceed absorption capacity, which leads to increased fecal moisture loss (Wu et al., 2023). Mucin 2 (Muc2) is a major glycoprotein that constitutes the intestinal mucus barrier, preventing microbial translocation through small pores (Ouyang and Xu, 2023). Research indicates that certain symbiotic bacteria can degrade or metabolize Muc2, resulting in reduced Muc2 expression, thinning of the intestinal mucus barrier, and exposure of epithelial cells directly to a disturbed intestinal microbiota environment, which is characteristic of intestinal diseases marked by fluid imbalance (Hansson, 2020). Ion channels and transporters associated with fluid metabolism, which are distributed among intestinal epithelial cells, serve as targets in fluid regulation, whereas the intestinal microbiota characteristic of diarrhea reflects the evolution of homeostasis. Mice with a knockout of the sodium-hydrogen exchanger 3 (NHE3) gene are more susceptible to chronic diarrhea; however, after treatment with Shenling Baizhu powder, the mRNA and protein expression of sodium−glucose cotransporter 1 (SGLT1) and NHE3 increased. SGLT1 also helps prevent LPS-induced apoptosis of intestinal epithelial cells, disruption of tight junctions, and increased intestinal permeability, thereby repairing intestinal mucosal epithelial cells during diarrhea (Yu et al., 2006; Peritore-Galve et al., 2023). Mice lacking cystic fibrosis transmembrane conductance regulator (CFTR) exhibit insufficient intestinal fluid secretion and constipation, yet an increase in *Bifidobacteria* is observed. Following drug treatment, the abundance of *Akkermansia* significantly increases, possibly affecting host mucosal anti-inflammatory pathways and enhancing epithelial integrity or correlating with increased expression of the intestinal antimicrobial peptide RegIII [see (Everard et al., 2013; Caesar et al., 2015; Ooi et al., 2018)].

### 5.3 Relationship between fluid metabolism and kidney level

Modern medicine recognizes the kidney as a central component of fluid metabolism that is closely linked to neuroendocrine, immune, reproductive, growth, developmental, and aging functions. The glomerular filtration rate and glomerular reabsorption function are the two primary metrics for assessing kidney function and evaluating urine circulation (Liu HY. et al., 2023). Clinical studies have shown that diarrhea is a common adverse effect among kidney transplant patients receiving drug treatment, particularly with azathioprine, which significantly reduces the glomerular filtration rate (Sekmek et al., 2021). Arginine vasopressin (AVP), synthesized in the thalamus, binds to vasopressin receptor type 2 (V2R) in renal collecting duct cells, activating protein kinase A (PKA) to regulate aquaporin (AQP) abundance (Feraille et al., 2022). AQPs modulate the movement of liquid and certain neutral small molecules in and out of cells, facilitating fluid reabsorption in proximal renal tubules and collecting ducts. Studies indicate that glycoproteins encoding liquid and glycerol transport via AQP3 cDNA are expressed in the kidney, urethra, bladder, and digestive tract. A compensatory relationship exists among certain aquaporins; mice with a double gene knockout exhibit greater impairment in urine concentration than those with a single AQP3 knockout (Ma et al., 2000). Moreover, the absorption of electrolytes and water in mammalian lumens primarily depends on NHEs and ENaCs, with hormones involved in renal secretion closely linked to fluid metabolism [see (Heilmann et al., 2023)]. Elevated serum aldosterone (ALD) levels in patients with IBD suggest that when ENaC, NHE3, and Na/K-ATPase are upregulated by ALD, a feedback mechanism mediated by the RAAS is present (Zaika et al., 2013; Rossier et al., 2015). Insufficient ALD secretion may exacerbate symptoms such as polyuria. Antidiuretic hormone (AVP) binds to G protein-coupled receptor type II (AVPR2), increasing cAMP levels and altering intracellular signaling, thereby influencing AQP expression (Xiong et al., 2019). In the pathological processes of certain diseases, downregulation of AVP expression or the levels of AQP2 and AVPR leads to a deficiency in the corresponding hormones or receptors, impairing the urine concentration and resulting in excessive excretion (Kwon et al., 2013; Moeller et al., 2016).

## 6 The mediating role of TCM in the renal-intestinal-microbiota axis to achieve “promoting urination to regulate bowel movements”

**Figure 5:**
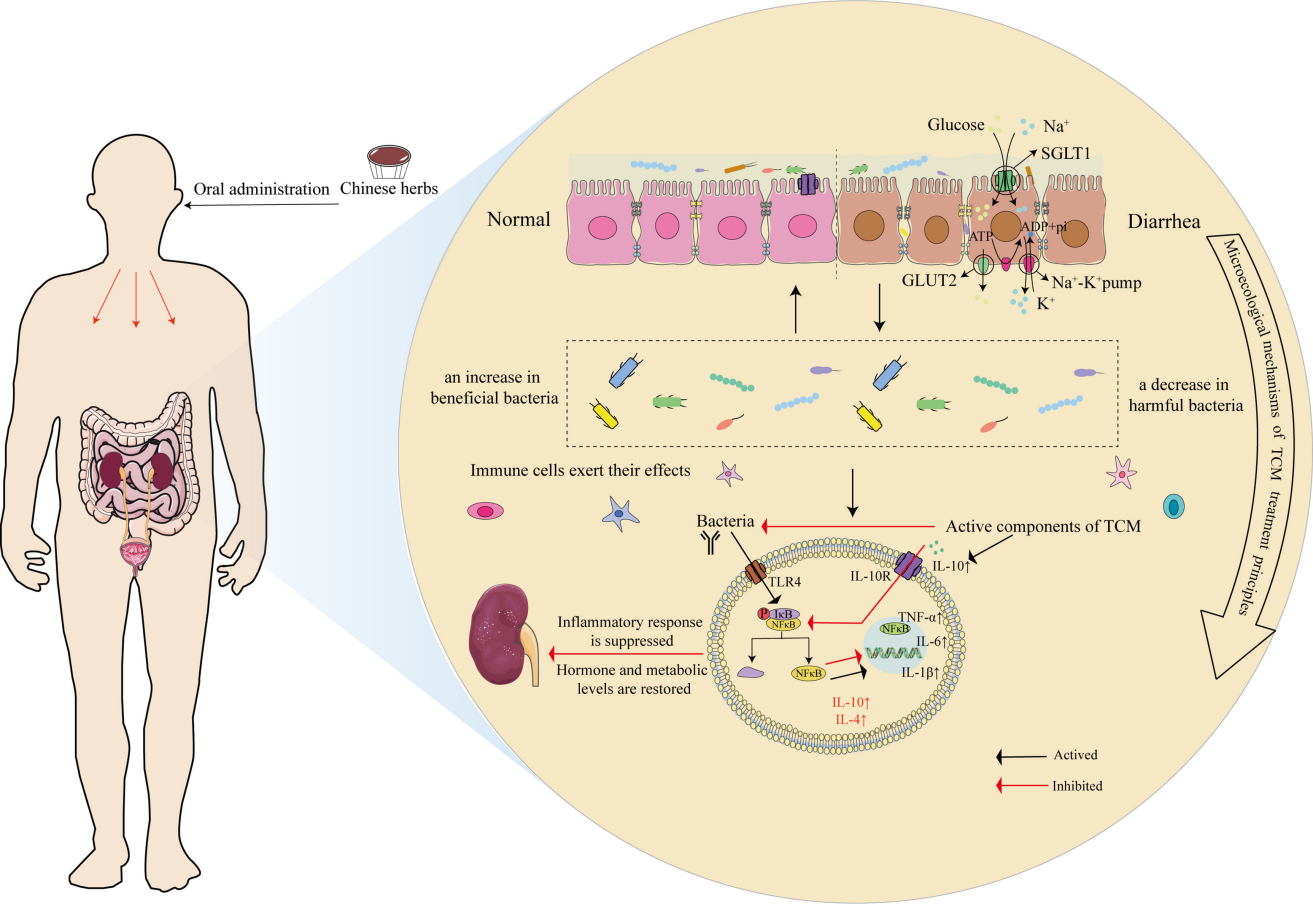
Microscopic mechanism of TCM in the treatment of diarrhea through the principle of “Promoting Urination to Regulate Bowel Movements.” Using the inflammation-related TLR4-NF-κB pathway as an example, after oral administration of Chinese medicine, it first affects the ion channels and transport proteins of mucosal epithelial cells, such as SGLT1 and GLUT2, and improves mucin production. Second, medicine increases the abundance of beneficial bacteria while reducing the proportion of harmful bacteria, thus regulating the intestinal microecology. Finally, the pharmacological effects act on bacterial LPS receptors (TLR4), inhibiting the binding of p-IκB/NF-κB proteins in their inactive state through specific signaling pathways, which subsequently releases NF-κB. NF-κB translocates to the nucleus, where it activates the transcription of target genes, resulting in increases in the levels of proinflammatory cytokines, such as TNF-α, IL-6, and IL-1β, whereas anti-inflammatory cytokines, such as IL-10 and IL-4, are activated to promote immune cell activity, mediate the activation, proliferation, and differentiation of T and B cells.

TCM encompasses multiple targets and pathways that regulate various mediators and signaling routes to intervene in target organs, restore homeostasis, and improve pathological states. The essence of the treatment principle of promoting urination to regulate bowel movements lies in the reregulation of fluid metabolism, with the intestinal microbiota and its metabolites participating in this process through the renal-intestinal axis. TCM formulae exemplified by “promoting urination to regulate bowel movements” warm and supplement the kidney yang, tonify the spleen, and transform dampness, mediating active components through neurotransmitters, neurotrophic factors, the RAAS, and the sympathetic nervous−adrenal axis to establish dynamic feedback within the mediator−microbiota network between the renal and intestinal axis (He et al., 2019; Zhou et al., 2024), as shown in **Table 1**, **Figure 5**).

## 7 Conclusion

TCM posits that the small intestine is responsible for governing transforming and thick body fluids, separating clear from turbid, while the kidney opens into the urethra and anus and governs water and fluids. Both organs play vital roles in fluid metabolism and the formation of both urine and feces. The essence of the treatment principle of “promoting urination to regulate bowel movements” lies in the redistribution of water and the management of fluid disturbances. Modern medicine highlights the critical roles of hormones and aquaporins in this process. The advent of microbiology provides a novel perspective for bridging the theories of TCM and modern medicine. With advancements in immunology, molecular biology, and medical statistics, the renal-intestinal axis network system is gradually elucidating the interactions betw. Simultaneously, the intestinal microbiota and their metabolic products function as mediating factors or antigens. Currently, most research utilizes the renal-intestinal axis to explore the potential for treating kidney disease through intestinal therapies and vice versa. However, the relationships between hormone levels related to liquid metabolism within the context of the renal-intestinal axis and microbiological mechanisms remainsremain unclear. This article examines the biological implications of the TCM treatment principle of “promoting urination to regulate bowel movements” through the impact of the intestinal microbiota and its metabolites on the renal-intestinal axis, elucidating the impact of the intestinal microecology on the biological mechanisms of fluid metabolism. Future studies will further investigate the correlation between specific intestinal microbiota and their metabolic products corresponding to different syndromes and their effects on the body’s hormones, channel proteins, and transporters. The goal is to provide a novel interpretation of the treatment principle of “promoting urination to regulate bowel movements” by utilizing the “renal-intestinal-microbiota” network system.
